# G-quadruplexes formation within the promoter of TEAD4 oncogene and their interaction with Vimentin

**DOI:** 10.3389/fchem.2022.1008075

**Published:** 2022-09-15

**Authors:** Marta Cozzaglio, Silvia Ceschi, Elisabetta Groaz, Mattia Sturlese, Claudia Sissi

**Affiliations:** ^1^ Department of Pharmaceutical and Pharmacological Sciences, University of Padova, Padova, Italy; ^2^ KU Leuven, Rega Institute for Medical Research, KU Leuven, Medicinal Chemistry, Leuven, Belgium

**Keywords:** G-quadruplex, TEAD4, Vimentin, G4-repeats, EMT

## Abstract

G-quadruplexes (G4s) are nucleic acid secondary structures detected within human chromosomes, that cluster at gene promoters and enhancers. This suggests that G4s may play specific roles in the regulation of gene expression. Within a distinct subgroup of G-rich domains, the formation of two or more adjacent G4 units (G4-repeats) is feasible. Recently it was shown that Vimentin, a protein highly expressed within mesenchymal cells, selectively recognizes these arrangements. Putative G4-repeats have been searched within the human gene proximal promoters by the bioinformatics tool QPARSE and they resulted to be enriched at genes related to epithelial-to-mesenchymal transition (EMT). This suggested that Vimentin binding at these sites might be relevant for the maintenance of the mesenchymal phenotype. Among all the identified sequences, in the present study we selected the one located within the promoter of the TEAD4 oncogene. TEAD4 codifies for a transcriptional enhancer factor, TEAD4, that actively promotes EMT, supporting, cell proliferation and migration. Moreover, in colorectal cancer cells TEAD4 directly enhances the expression of Vimentin. Thus, the possible interaction of Vimentin with TEAD4 promoter could highlight a positive feedback loop between these two factors, associated to important tumor metastasis related events. Here, we exploited spectroscopic and electrophoretic measurements under different conditions to address the folding behavior of the selected sequence. This allowed us to validate the folding of TEAD4 promoter into a G4-repeat able to interact with Vimentin.

## 1 Introduction

Starting from 1953, when Watson and Crick identified the B-form of the DNA (Watson et al., 1974), up to our days, it has been clarified that DNA is a very plastic molecule, that can fold into a variety of secondary structures (Bochman et al., 2012). G-quadruplex (G4) is a non-canonical DNA secondary structure, which corresponds to a tetrahelical arrangement that can form at guanine-rich tracts. Here, four guanine residues interact through Hoogsteen hydrogen bonds and give rise to planar arrays, called G-tetrads, that interact with each other through π-π stacking. This structural core is further stabilized by monovalent cations that are coordinated in the center of the tetrads (Largy et al., 2016). The four strands of the G4 may belong to just one (monomolecular arrangement) or different (bi- or tetra-molecular arrangements) DNA filaments. In both instances, the strands can pair with variable relative orientations, based on which G-quadruplexes can be classified into parallel, antiparallel, or hybrid.

To fold into a monomeric G4 structure, a DNA filament must contain four runs of at least two consecutive guanines, each of them separated by no less than one nucleotide. Based on these sequence requirements, several algorithms have been designed to identify those regions potentially capable of forming G4 structures within the genome (Todd 2005). These sites, defined as PQS (potential G4 forming sequences) are enriched mainly at telomeres, gene promoters, ribosomal DNA, recombination sites and 5′-UTR regions (Rigo et al., 2017). In living cells, the presence of G4s at these sites has been experimentally confirmed thanks to the development of antibodies and fluorescent probes capable of selectively recognizing these structures (Schaffitzel et al., 2001). These analyses associated multiple physiological roles of G4s to their genomic location. In particular, the enrichment in PQS around the TSS (transcription starting site) of various oncogenes suggested an important role of G4 structures in the regulation of their expression (Eddy and Maizels 2006). This was further confirmed by the ability of G4 selective ligands to influence the expression of their target genes (Balasubramanian 2011).

More recently, the interest was attracted by those sites where multiple PQS are located in close positions along the genome since this clustering was not the simple result of a statistical enrichment of guanines at these sites (Berselli et al., 2019). Noteworthy, this PQS distribution can support the folding of multiple G-quadruplex units within a single short DNA fragment, giving rise to the so-called G4-repeats. The first *in vitro* structural studies of G4-repeats were based on the human telomeric sequence and later on they were extended to oncogene promoters such as hTERT, ILPR, KRAS, c-KIT and c-MYC (Monsen et al., 2021), (Monsen et al., 2020), (Schonhoft et al., 2009), (Marquevielle et al., 2020), (Rigo and Sissi 2017), (Monsen et al., 2022).

Recently, it has been observed that Vimentin, an intermediate filament (IF) protein, is able to interact selectively with G4-repeats. This complex involves only the tetrameric form of the protein that is found within the nucleus as a soluble nuclear fraction (Ceschi et al., 2022). Although an architectural role has long been considered the main function of Vimentin, it is well recognized that it also participates to various signaling cascades and it can act as a transcriptional regulator within metastatic cancer cells (Mergui et al., 2010), (Jang et al., 2021). In particular, Vimentin expression is highly reactivated during epithelial to mesenchymal transition (EMT), a process that occurs during both physiological tissue development/regeneration and pathological cancer progression towards metastasis. Noteworthy, a bioinformatic search for G4-repeats at proximal gene promoters, followed by GO analysis, highlighted an intriguing correlation between the functions of Vimentin and those of the genes containing putative Vimentin binding sites at their promoters (Ceschi et al., 2022).

Among all the genes identified as potentially able to accommodate a G4-repeat at their proximal promoter, we were interested in TEAD4. This gene, located at chromosome 12, codifies for a transcriptional enhancer factor (TEAD4) that interacts with YAP/TAZ and other transcription factors to regulate different cellular processes. Among them, TEAD4 plays an important role in cancer, being actively involved in the regulation of metastatic behavior and cancer stem cell dynamics (Barry. 2021) (Chen et al., 2020). Remarkably, it has been shown that in colorectal cancer cells TEAD4 promotes EMT in a YAP independent manner, regulating the expression of Vimentin (Liu et al., 2016).

In the present study, we evaluated the structural features of the G4-repeat putatively occurring at the TEAD4 proximal promoter and we addressed its interaction with Vimentin. These data will help to clarify whether the recruitment of Vimentin at G4-repeats might represent a mechanism through which the regulation of different EMT-related genes such as TEAD4 is achieved.

## 2 Materials and methods

### 2.1 Oligonucleotides

Oligonucleotides were purchased from Eurogentec (Liége, Belgium) as RP-HPLC purified products. Oligonucleotides were dissolved in milliQ H_2_O to prepare 100 µM stock solutions. Before use, each sample was heated at 95°C for 7 min in the required buffer and then slowly cooled down at room temperature to equilibrate the system. The DNA sequences used in this work are listed in Table 1.

**TABLE 1:** DNA sequences herein studied.

Sequence name	Sequence reported in the 5′-3′ direction
TEAD4-full	CGG​GCG​GGC​GAG​GGG​CCG​GGC​CGC​CGG​GGC​GGG​GCG​GGG​CCG​GGC
TEAD4-full-2TT	CGG​GCG​GGC​GAG​GGG​CCG​GGT​TGT​TGG​GGC​GGG​GCG​GGG​CCG​GGC
TEAD4-far	CGG​GGC​GGG​GCG​GGG​CCG​GGC
TEAD4-near	CGG​GCG​GGC​GAG​GGG​CCG​GGC
Marker 22 nts	GGA​TGT​GAG​TGT​GAG​TGT​GAG​G
Marker 44 nts	GGA​TGT​GAG​TGT​GAG​TGT​GAG​GGG​ATG​TGA​GTG​TGA​GTG​TGA​GG

The ability of tested oligonucleotides to form intramolecular hairpins was predicted by the use of the IDT OligoAnalyzer tool (https://eu.idtdna.com/calc/analyzer). Setting parameters were 150 mM Na^+^ and 2 µM oligo concentration.

### 2.2 Circular dichroism

Circular dichroism spectra were recorded on a Jasco J-810 spectropolarimeter equipped with a Peltier temperature controller using 1 cm pathlength quartz cuvette. CD spectra were recorded from 230 to 330 nm. DNA samples were used at 2–4 μM final concentration in 5 mM Tris, pH 7.5. The salt concentration and the temperature used for the experiments varied according to the purpose of the assays.

For CD kinetic experiments, spectra were acquired immediately after the manual addition of the selected cation into the cuvette from a stock solution, with the following parameters: scanning speed 200 nm/min; interval scan of 60 s, bandwidth of 2 nm; data interval of 0.5 nm; response of 1 s.

For CD melting experiments, spectra were acquired between 95 and 25°C every 2°C. Each spectrum represents the average of 2 scans acquired with the following parameters: scanning speed 100 nm/min; band width of 2 nm; data interval of 0.5 nm; response of 2 s. Spectra were reported as molar ellipticity ([θ]). To analyze the variation of signals at single wavelength each data point was subtracted of the starting CD value contribution and the difference was normalized between 0 and 1 (relative molar ellipticity).

### 2.3 Thermal differential spectra (TDS)

Thermal differential spectra were obtained by subtracting the oligonucleotide UV-spectrum acquired at 25°C from the one recorded at 95°C. Experiments were performed using 2–4 µM oligonucleotide solution in 5 mM Tris pH 7.5, 150 mM KCl.

### 2.4 Expression and purification of vimentin

The genomic sequence that corresponds to full-length human Vimentin (residues Met1-Glu466) was inserted into the pET21a + vector by Genescript. The plasmid was used to transform competent *E. Coli* BL21 (DE3) cells. Bacteria were grown at 37°C in LB media until an OD_600_ of 0.7 was reached. At this point, the expression of Vimentin was induced by adding 1 mM isopropyl-β-d-thiogalactopyranoside (IPTG). Vimentin was purified as previously described (Herrmann et al., 2004). Briefly, cells were collected by centrifugation at 7000 g for 20 min at 4°C, resuspended in 10 mM Tris, 2 mM PMSF, 10 mM MgCl_2_, pH 7.5. After resuspension, the cells were sonicated and up to 0.2% Triton X-100 and 50 g/ml DNase I were added. The inclusion bodies were harvested by centrifugation, and then resuspended in the corresponding washing buffer (10 mM Tris, 1 mM EDTA, 1 mM DTT, 2 mM PMSF, 1.5 M KCl, pH 7.5). After centrifugation, this procedure was repeated with the same buffer without KCl. Finally, the pellets were washed with 10 mM Tris, 0.1 mM EDTA, pH 7.5. After the last washing step, inclusion bodies were resuspended in a denaturing buffer (9.5 M urea in 10 mM Tris, pH 7.5). The solubilized IF proteins were collected by centrifugation at 60,000 g for 1 h at 20°C. Vimentin was purified from the supernatant by two steps of exchange chromatography, using first an anionic (Q Sepharose FF, Cytiva) then a cationic (SP Sepharose FF, Cytiva) column. The buffer used for the exchange chromatography was 10 mM Tris, 500 mM NaCl, 1 mM DTT, pH 7.5. Purified Vimentin was stored at −80°C.

### 2.5 Protein sample preparation

The day before use, Vimentin was renatured following the protocol developed by Herrmann and others to avoid polymerization into filaments (Herrmann et al., 2004). The protein was dialyzed at room temperature against dialysis buffer (5 mM Tris, 1 mM EDTA, 0.1 mM EGTA, 1 mM dithiothreitol, pH 8.4) containing a progressively reduced urea concentration (6, 4, 2, and 1 M urea). Dialysis against a large volume of buffer with 1M urea proceeded overnight at 4°C. The next day, dialysis was continued against tetramer buffer (5 mM Tris, pH 8.4) for 1 h at room temperature. After dialysis, the protein concentration was determined by measuring the absorption at 280 nm with *ε* = 24,900 cm^−1^M^−1^.

### 2.6 Polyacrylamide gel electrophoresis

For each sample, a solution of oligonucleotide (2–4 μM strand concentration) in 5 mM Tris, pH 7.5 was heated at 95°C for 7 min and allowed to cool down to room temperature overnight. Afterwards, variable concentrations of KCl (0, 10 and 150 mM) were added to the samples and they were led to equilibrate overnight at room temperature. The samples were then loaded on a native 15% polyacrylamide (19:1 acrylamide: bis-acrylamide) gel in 1 × TBE (89 mM Tris, 89 mM boric acid, 0.02M EDTA) implemented with 20 mM KCl. The gel was stained with Sybr Green II and the resolved bands were visualized on an image acquisition system (Geliance 600 Imaging system, Perkin-Elmer).

### 2.7 Electrophoretic mobility shift assays

Electrophoretic mobility shift assays (EMSA) were performed on 1.5% agarose gels in 1 × TBE buffer and 10 mM KCl. Each reaction sample contained 500 nM DNA and 8 μM Vimentin, in 5 mM Tris, 150 mM KCl, pH 8.4. Vimentin was added to oligonucleotide solutions and incubation was performed for 1 h at room temperature. Immediately before loading, 3 μl of gel loading buffer (50% glycerol, 50% water) were added to the samples. Electrophoresis proceeded for 2 h at 6 V/cm at 4°C. Gels were stained with Sybr Green II to visualize nucleic acids and with Coomassie Brilliant Blue G250 for protein detection. Gel images were acquired with a Geliance 600 apparatus.

## 3 Results

### 3.1 Rational selection of a sequence that potentially folds into a G4-repeat

In a previous study (Ceschi et al., 2022) the Q-PARSE bioinformatics tool was used to search sequences containing two contiguous G4-forming elements in the region covering 100 nucleotides upstream the transcription starting site of human genes. The search was extended to both, the forward and reverse strands. The searched pattern comprised islands of 3–4 guanines with connecting loops of maximum 5 nucleotides. The software provided an output of 1477 genes that contain at least one putative double G4-repeat. Among the retrieved sequences, we chose to characterize a sequence, TEAD4-full (Table 1), located 31 nucleotides upstream the TSS of the TEAD4 oncogene.

**FIGURE 1:**
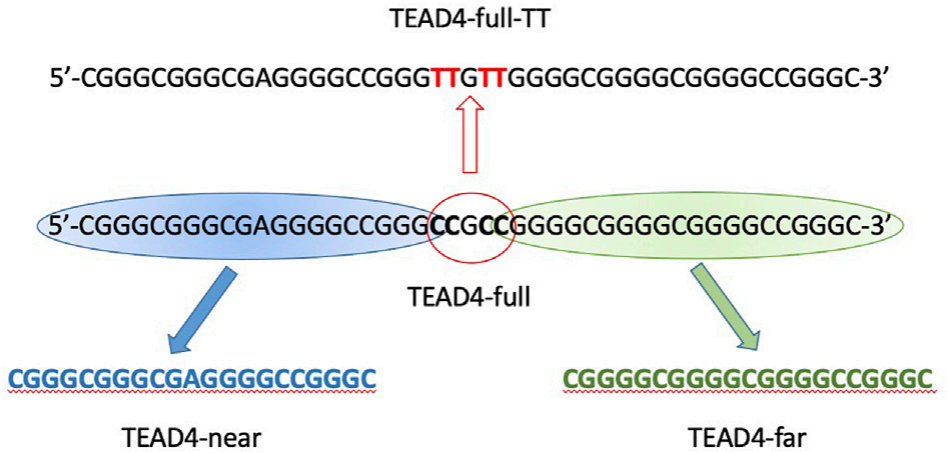
Schematic representation of TEAD4 studied sequences.

Besides the functional roles of the protein encoded by this gene, the selected sequence represented an attracting system thanks to its guanines number and distribution. The study of any G4-repeat system might be very complex due to the potential coexistence of multiple structural equilibria (Monsen et al., 2022). However, it was possible to subdivide TEAD4-full into two shorter domains, each of them comprising 4 runs of at least 3 guanines (thus each of them putatively able to fold into a stable G4), separated by a short linker (Figure 1). Thus, we took advantage of this base composition of TEAD4-full and we started its characterization by analyzing the conformational features of the two short sequences located at the 5′- and 3′-end, addressed as TEAD4-near and TEAD4-far, respectively.

### 3.2 TEAD4-near folds prevalently into an antiparallel G-quadruplex structure

**FIGURE 2:**
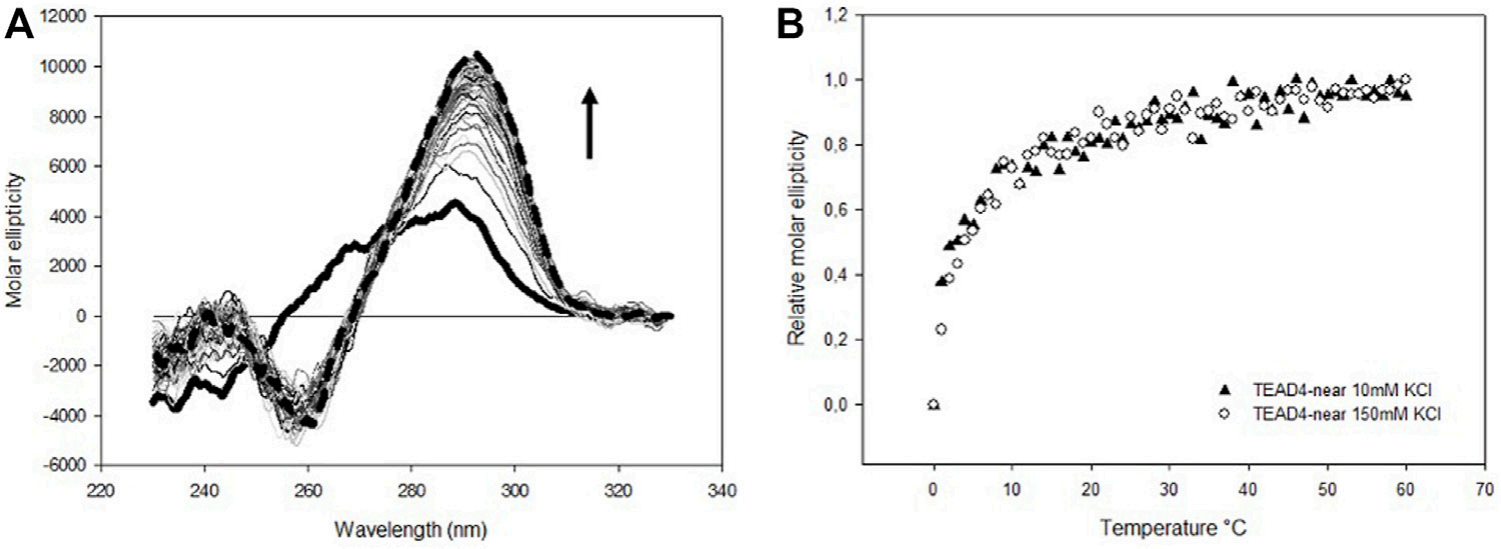
**(A)** Time dependent variation of CD spectra of 4 µM TEAD4-near in 5 mM Tris pH 7.5, upon addition of 150 mM KCl recorded at 25°C. Solid and dashed lines correspond to the oligonucleotide in the absence of metal ion and after 1 h incubation, respectively. **(B)** Time dependent relative variation of molar ellipticity at 290 nm of 4 µM TEAD4-near in 5 mM Tris pH 7.5, upon addition of 10 or 150 mM KCl.

To address the conformational features of the selected sequences, we performed CD spectroscopy that allows to follow the folding process of oligonucleotides and provides useful insights into the topology of the nucleic acid arrangements in solution. Since it is known that G-quadruplex formation is promoted by the presence of some cations, in particular potassium, we started our analyses by monitoring the modifications of the CD spectrum of TEAD4-near in 5 mM Tris, pH 7.5 upon addition of 10 and 150 mM KCl (Largy et al., 2016). Under both conditions, we observed an increase in molar ellipticity at 290 nm and a decrease at 260 nm that occurred in the same time scale at the two tested KCl concentrations (Figure 2).

The shape of the CD spectrum is known to be different for a G4 of parallel or antiparallel topology (Balagurumoorthy et al., 1992). Parallel G4s typically show a positive signal at 265 nm and a negative one at 240 nm, whereas antiparallel quadruplexes exhibit a positive signal at 290 nm and a negative one around 260 nm. Thus, the CD spectra of TEAD4-near suggested that the addition of potassium drove the formation an antiparallel G4. The presence of a G4 structure was further confirmed by the UV thermal differential spectrum (TDS) (Supplementary Figure S1A), that exhibited the characteristic pattern associated to this non-canonical structure, with two positive peaks at 240 and 277 nm, a shoulder at 255 nm and a negative minimum at 296 nm (Mergny 2005).

**FIGURE 3:**
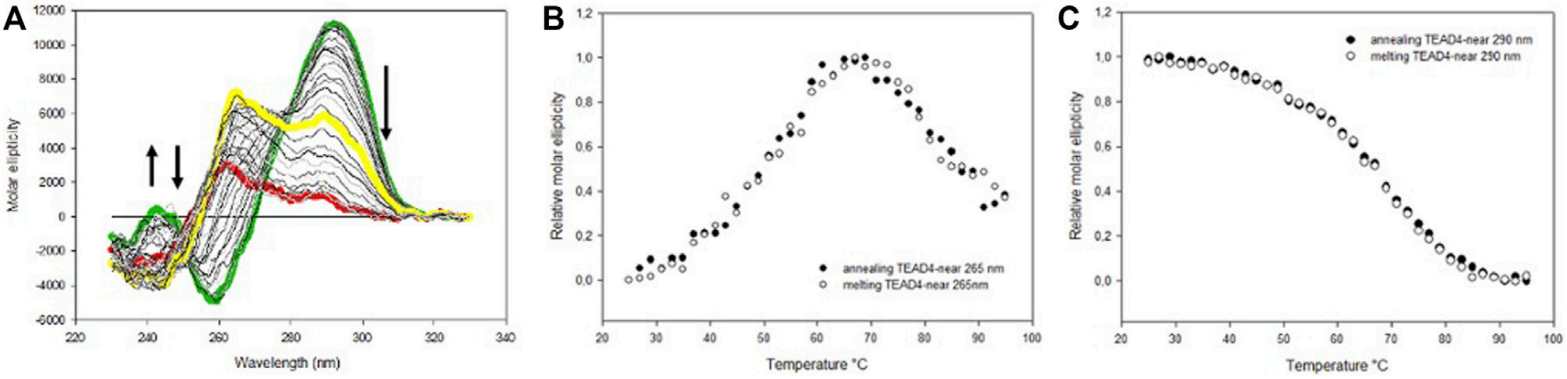
**(A)** CD spectra of 4 µM TEAD4-near in 5 mM Tris pH 7.5, 150 mM KCl, acquired at increasing temperatures. The green, yellow and red lines correspond to spectra acquired at 25, 70 and 95°C, respectively. **(B)** and **(C)** Variation of the relative molar ellipticity at 265 and 290 nm, respectively, recorded during the annealing and the melting of 4 µM TEAD4-near in 5 mM Tris pH 7.5, 150 mM KCl.

The melting and annealing profiles of TEAD4-near were acquired at the physiologically relevant KCl concentration (150 mM) and they were perfectly reversible (Figure 3). The melting profile showed a progressive reduction of the CD signal at 290 nm. This decrease was paired with an increment of the signal at 265 nm that reached the maximal intensity at about 70°C and that was lost at higher temperatures (Figure 3A). This transition can be attributed to an intermediate state occurring along the folding/unfolding of the antiparallel G4 but which presence was negligible at physiological temperature.

To verify whether some of the detected species might correspond to intermolecular arrangements, native polyacrylamide gel electrophoresis (PAGE) experiments were performed. TEAD4-near samples were analyzed before and after an annealing step carried out in the presence of KCl. All of them run as a single band with an electrophoretic mobility higher compared to the one of an unfolded 22-nts scrambled oligonucleotide (Supplementary Figure S2A), thus confirming that TEAD4-near folds only into intramolecular structures under our experimental conditions.

### 3.3 The folding of TEAD4-far comprises parallel and antiparallel G4 components

**FIGURE 4:**
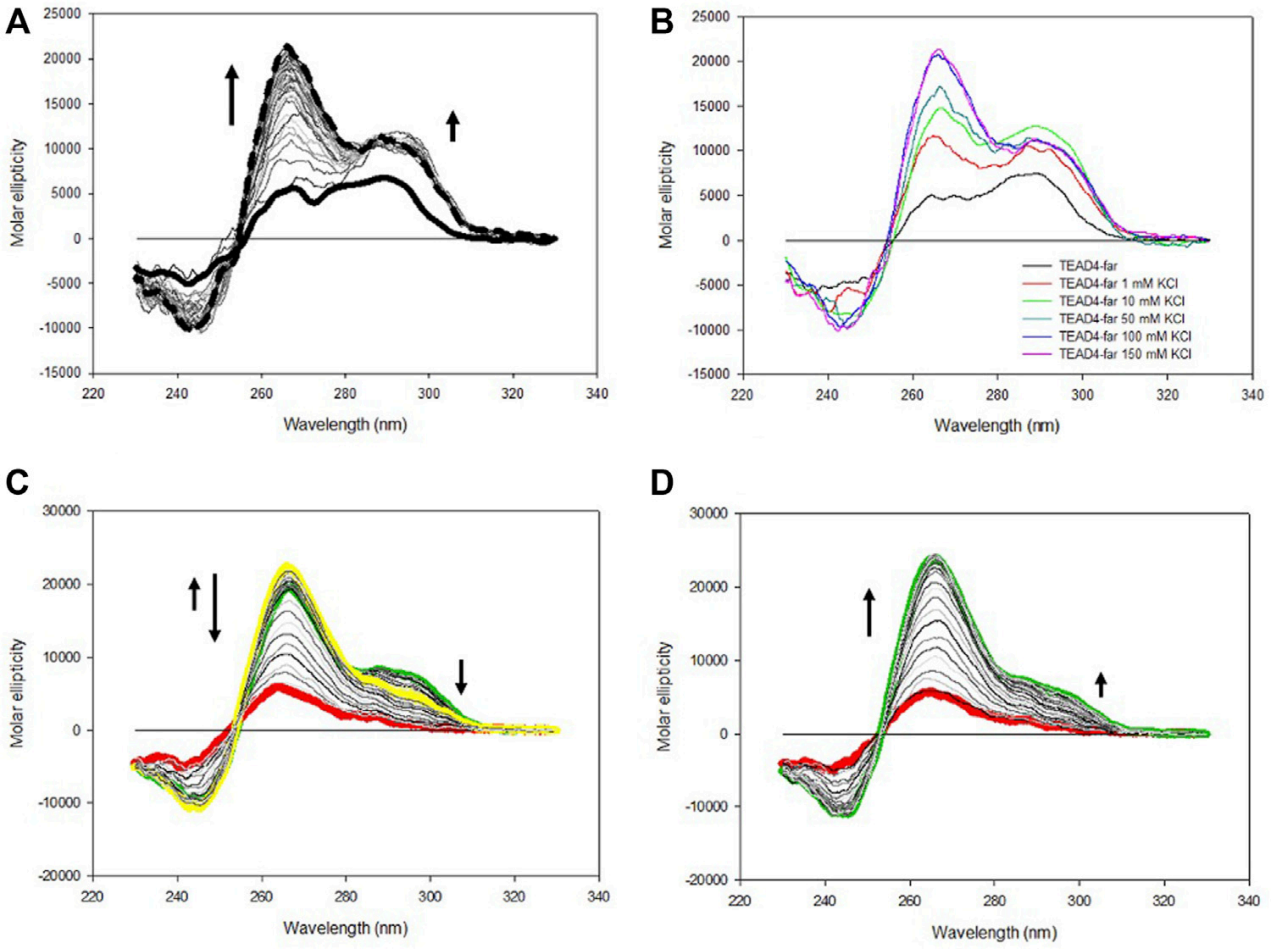
**(A)** Time dependent variation of CD spectra of 4 µM TEAD4-far in 5 mM Tris, pH 7.5 upon addition of 150 mM KCl at 25°C. Solid and dashed lines correspond to the oligonucleotide in the absence of metal ion and after 1 h incubation, respectively. **(B)** CD spectra of 4 µM TEAD4-far in 5 mM Tris pH 7.5 in the absence and presence of 1–150 mM KCl acquired after 1 h at 25°C. **(C)** and **(D)** CD spectra of 4 µM TEAD4-far in 5 mM Tris pH 7.5, 150 mM KCl, acquired during melting **(C)** or annealing **(D)** at 20°C/h. The green, yellow and red lines correspond to spectra acquired at 25, 65 and 95°C, respectively.

Consistently with the above described experimental approach, we started the characterization of TEAD4-far structural equilibria by monitoring in time the CD spectra modifications upon addition of increasing KCl concentrations up to 150 mM (Figure 4A). At all tested conditions, the CD spectra of TEAD4-far progressively evolved towards the formation of two main positive peaks located at 290 and 265 nm that, as supported by TDS, were associated to its folding into G4 (Supplementary Figure S1B).

CD spectra with maxima at both 265 and 290 nm are typical of G4 with hybrid topology (Balagurumoorthy et al., 1992). Alternatively, they can be observed when a sequence in solution distributes between parallel and antiparallel conformations. Here, by comparing the molar ellipticity of the oligonucleotide solutions after 1 h of incubation, it emerged that their relative intensities were a function of the metal ion concentration (Figure 4B). As far it concerns the peak at 265 nm, it reached the maximum of molar ellipticity at 100 mM KCl. Conversely, the intensity of the peak at 290 nm increased moving from 1 to 10 mM KCl while further increments of the metal ion concentration reduced it to converge towards a constant value of molar ellipticity. The differential response at these two wavelengths was further highlighted by comparing the folding kinetic of the oligonucleotide at these two wavelengths, the variation of the signal at 265 nm being much slower than the one occurring at 290 nm at all tested KCl concentrations (Supplementary Figure S3). These data indicated a competition between two distinct oligonucleotide arrangements that leads to their different relative abundance as a function of KCl concentration. At high KCl concentration parallel G4 arrangement was favored. Interestingly, melting/annealing profiles acquired in 10 mM KCl nicely overlapped (Supplementary Figure S4). Conversely, in 150 mM KCl, by heating of the solution up to 65°C, a modest but significant rearrangement occurred, which corresponded to an increase of the molar ellipticity at 265 nm associated to a reduction of the signal intensity at 290 nm (Figures 4C,D). Noteworthy, this species was the one preserved at r.t. after the annealing.

Overall, data suggested that TEAD4-far distributes between a kinetically favored antiparallel structure and a thermodynamic favored parallel conformation, the annealing shifting the system towards the last one at physiologically relevant KCl concentrations. PAGE experiments confirmed that none of them is related to intermolecular folded structures (Supplementary Figure S2B).

### 3.4 TEAD4-full folds into an extended parallel G4

**FIGURE 5:**
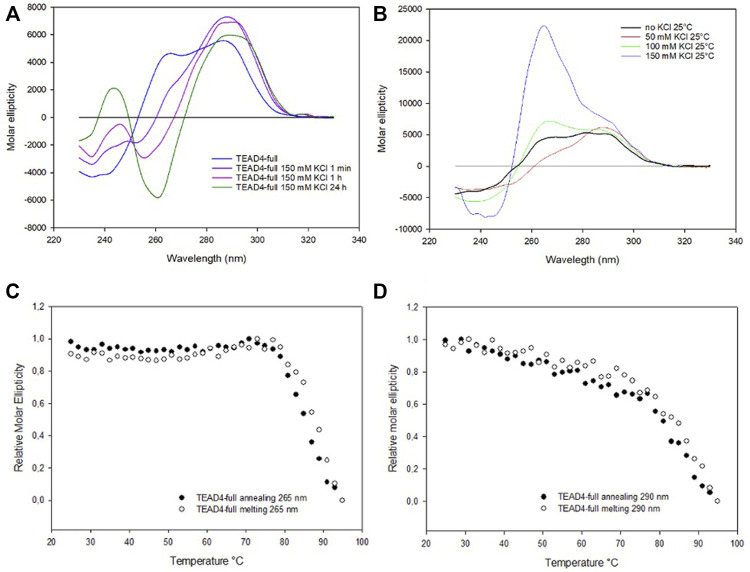
**(A)** CD spectra of 2 µM TEAD4-full in 5 mM Tris, pH 7.5, in the absence of KCl and 1 min, 1 h and 24 h after the addition of 150 mM KCl at 25°C. **(B)** CD spectra of 2 µM TEAD4-full in 5 mM Tris after annealing in presence of 50, 100 or 150 mM KCl. **(C)** and **(D)** Relative variation of the molar ellipticity recorded at 265 and 290 nm, respectively, during annealing and melting of 2 µM TEAD4-full in 5 mM Tris pH 7.5, 150 mM KCl at 20°C/h.

Based on the above presented results, it was unexpected to observe that the addition of KCl up to 150 mM to TEAD4-full produced only modest changes of the dichroic signal. They comprised a fast increment at 290 nm that was paired with a very slow rearrangement at lower wavelengths that occurred on the time scale of hours (Figures 5A; Supplementary Figure S5). This chiroptical profile markedly changed as a function of potassium concentration when we analyzed the annealed oligonucleotide solutions (Figure 5B) with a positive band centered at 265 nm progressively emerging above 50 mM KCl. In particular, at 150 mM KCl, this contribution was largely dominant. UV thermal differential scans confirmed that it corresponded to TEAD4-full preferentially folded into G4, since the negative signal at 296 nm, characteristic of the G4 structure, was remarkably more intense when compared to the one acquired before the annealing step (Supplementary Figure S1C–D). These data supported the preferential formation of a parallel G4 at physiological KCl concentration.

**FIGURE 6:**
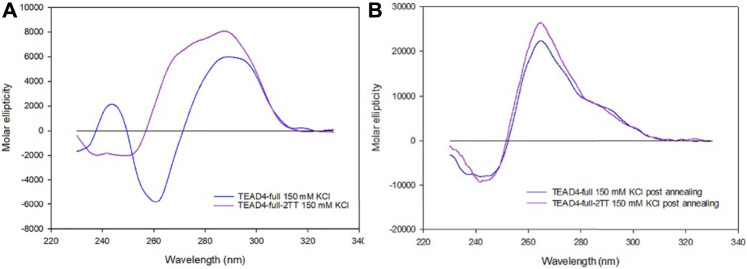
CD spectra of 2 µM TEAD4-full and TEAD4-full-2TT in 5 mM Tris, pH 7.5, 150 mM KCl acquired 24 h after addition of the salt at 25°C **(A)** or after annealing **(B)**.

Worth of note, once annealed, at all tested salt concentrations the system was at the thermodynamic equilibrium and, subsequent melting/annealing cycles were fully reversible (Figure 6). Their profiles further supported that at the lower tested KCl concentration the presence of multiple components was preserved (Supplementary Figure S6). Also for this sequence, PAGE confirmed the conserved presence of only intramolecular structures (Supplementary Figure S2C).

According to these data, it is possible to propose that the addition of metal ion to TEAD4-full stabilized an intramolecular arrangement that prevents the formation of the G4-repeat, the thermal denaturation of this starting arrangement(s) allowing the formation of the more thermodynamic stable parallel G4. Based on the intensity of the signal, it appeared that at physiological conditions it covered both the two G4 domains. This is compatible with the formation of a G4-repeat.

### 3.5 The mutated TEAD4-full-2TT adopts a parallel G4 conformation

The described structural folding landscape of TEAD4-full is complex. To better describe it, we designed a sequence with a reduced propensity to adopt G4-competing structures. By analyzing the TEAD4-full sequence with the IDT OligoAnalizer prediction tool, we retrieved several hairpin structures, as expected for a sequence with such a remarkable high GC content. Noteworthy, the most stable hairpins involved constantly the central four cytosines paired with different guanines that, based on our data on the short TEAD4 domains, should be involved in G-tetrads formation (Supplementary Figure S7). To validate the potential impact of these secondary structures on the G4 formation, we considered to reduce them by converting the central cytosines into thymines (Figure 1). Thus, we characterized the mutated sequence TEAD4-full-2TT reported in Table 1.

The CD spectra of TEAD4-full-2TT after the addition of 150 mM KCl showed a weak peak at 290 nm similar to the one of TEAD4-full. However, differently from the wild-type sequence, it comprised also a weak shoulder at 265 nm, likely due to the presence of a small fraction of parallel G4 (Figure 6A). The annealing of this solution readily drove a strong increment of this component, comparable to TEAD-full although slightly more intense (Figure 6B). These data indicated that the introduced mutations did not fully prevent the formation of potentially G4-repeat competing structures, but they reduced them thus making more efficient the structural rearrangement towards the thermodynamically favored parallel G4-repeat.

### 3.6 The G4-repeats of TEAD4-full and TEAD4-full-2TT are recognized by Vimentin

To address the binding of Vimentin to TEAD4, we performed electrophoretic mobility shift assays with the purified recombinant human protein. Binding reactions were carried out at pH 8.4 to avoid Vimentin polymerization into filaments (Herrmann et al., 2004). Due to the complex folding landscape of the herein tested sequences, oligonucleotides were either equilibrated or annealed in 150 mM KCl to promote G-quadruplex formation before protein addition.

**FIGURE 7:**
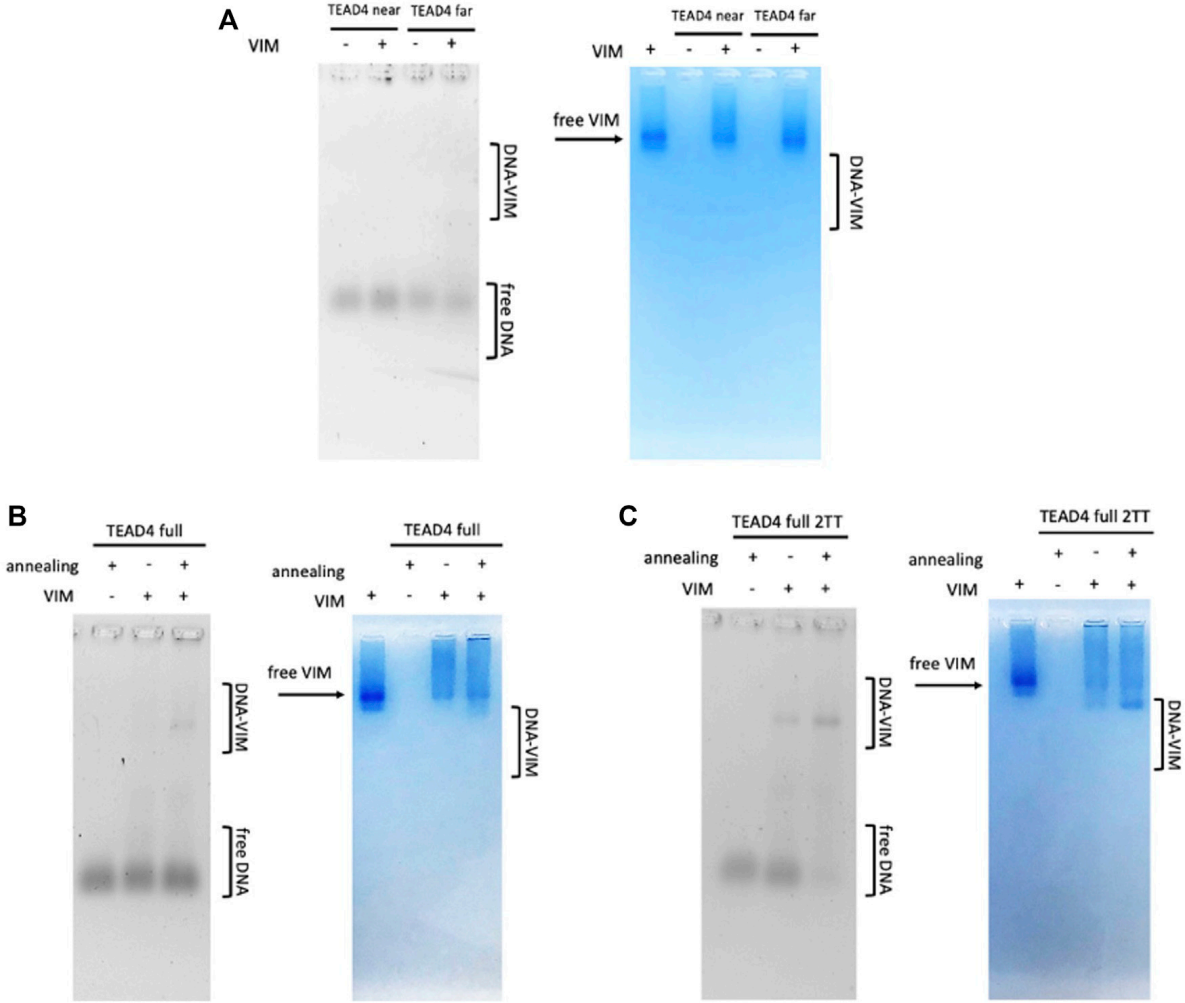
EMSA of tested oligonucleotides (500 nM) incubated with 8 µM Vimentin in 5 mM Tris, pH 8.4, 150 mM KCl, stained with Sybr Green II (on the left) and Coomassie Brilliant Blue G250 (on the right) Panel **(A)** TEAD4-near and TEAD4-far; Panel **(B)** TEAD4-full before and after annealing in presence of KCl; Panel **(C)** TEAD4-full-2TT before and after annealing in presence of KCl.

As expected from the high selectivity of Vimentin for G4-repeats, no protein-DNA complexes were observed when Vimentin was incubated with the isolated TEAD4-near and TEAD4-far (Figure 7A). Conversely, as shown in Figure 7B, Vimentin interacted with TEAD4-full. Remarkably, the complex formation occurred only when the oligonucleotide was previously annealed in 150 mM KCl. This result further confirmed that the parallel G4 arrangement induced in these conditions actually corresponds to a G4-repeat.

It was interesting to observe that Vimentin did bind to TEAD4-full-2TT either before and after the annealing. In the first case, the interaction was weak, while no free DNA was observed when Vimentin was added to the previously annealed TEAD4-full-2TT (Figure 7C). The higher affinity of the protein for this full-length mutated sequence further indicated that the selected mutations efficiently reduced the formation of structural motives that compete with the complete folding of TEAD4-full into a G4-repeat.

## 4 Discussion

TEAD4 is an oncogene with important roles in cancer, including epithelial-to-mesenchymal transition (EMT), metastasis, chemotherapeutic drug resistance and, consistently, it represents a valuable target for anticancer treatment (Chen et al., 2020). The presence of a long G-rich domain within its promoter is attractive since its potential conversion into non-canonical DNA arrangements may lead to unique sites of intervention to modulate its functions. This will potentially drive towards a better description of TEAD4 functional roles and eventually to novel therapeutic tools. As a starting point, it was demanding to explore the conformational profile of this G-reach domain.

The combined data obtained by CD and UV spectroscopic analyses at physiologically relevant KCl concentration, showed that at the identified long G-rich site, two short DNA domains can individually fold into different G4 structures. TEAD4-far folds into a combination of parallel and antiparallel intramolecular G4s, the antiparallel ones being kinetically favored, while the parallel ones being more thermodynamically stable and, remarkably, being largely dependent on KCl concentration. In principle, TEAD4-near can be addressed as less polymorphic since in solution it mainly folds into an intramolecular antiparallel G4. However, along the folding pathway, a parallel contribution was also detected. Although at physiological temperature its presence is negligible *in vitro*, we cannot rule out its occurrence to a significant extent in the complex nuclear environment.

**FIGURE 8:**
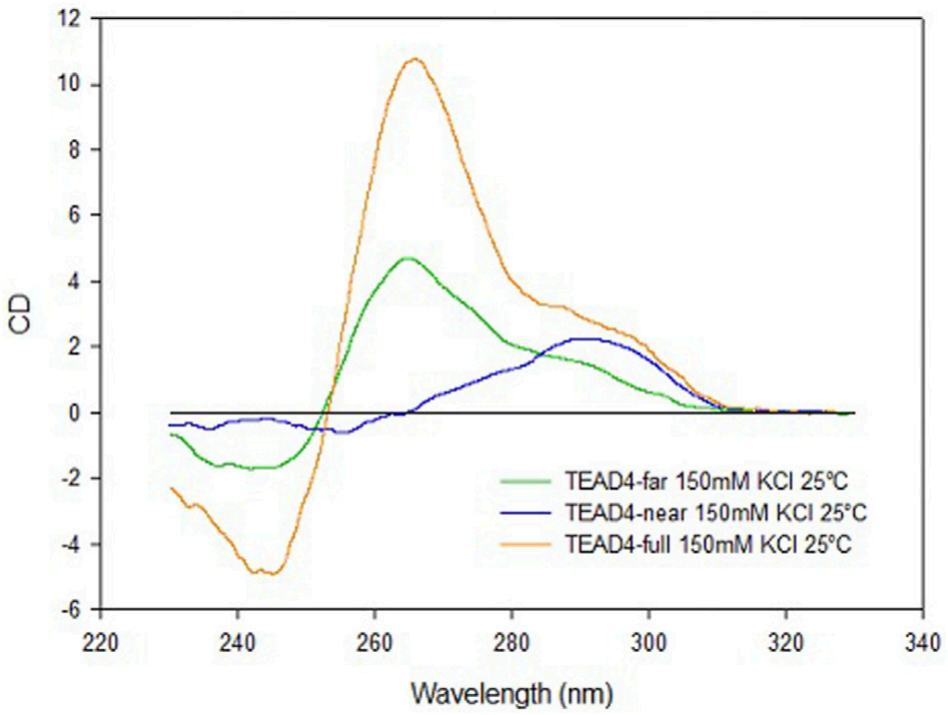
CD spectra of TEAD4-full, TEAD4-far and TEAD4-near in 5 mM Tris, pH 7.5, after annealing in presence of 150 mM KCl.

As supported by the interaction of TEAD4-full with Vimentin, the full-length G-rich domain folds into a repeat formed by two G4 modules. However, the folding behavior of the full-length sequence TEAD4-full, did not correspond to the sum of its two domains as clearly emerged by comparing their chiroptical contributions (Figure 8). In particular, it appears that TEAD4-near, being prevalently folded into an antiparallel G4, does not contribute to the relevant parallel G4 content (as derived from the positive band at 265 nm) of the full length TEAD4-full. This issue can be addressed based on literature data. Indeed, conformational rearrangements occurring when multiple G4 modules are arranged into a continuous array have been already highlighted, first of all for the telomeres, that can accommodate several nearby G4s with the same sequence composition but with a combination of distinct topologies (Monsen et al., 2021) (Carrino et al., 2021). More recent evidences pointed that at gene promoters, contiguous G4s are expected to evolve towards a conserved preferential parallel conformation (Schonhoft et al., 2009) (Monsen et al., 2022). It has been proposed that this can be the result of a favorable contribution deriving from G4-G4 stacking interaction, easily occurring between two parallel G4s but hindered with the antiparallel ones. In our model, as above mentioned, TEAD4-near can undergo an antiparallel to parallel transition, thus it is not at odd to consider that the last is preferred when it is flanked by the TEAD4-far.

The parallel G4-repeat of TEAD4-full is expected to be much more stable in comparison to the two isolated G4 units. However, we observed how its folding is impaired before the annealing at high KCl concentration. Here, as supported by the characterization of the mutated TEAD4-full-2TT sequence, we related this to the possible competition between G4-repeats and alternative intramolecular pairings. Again, this evidence does not rule out the formation of the G4-repeat in the intracellular environment where the complex composition can influence the relative stabilities of the folded species. Moreover, we should consider that in the cell, the herein studied sequence is inserted within a long frame of dsDNA. While as previously reported (Buglione et al., 2021) this template well supports G4-repeat formation, it might elicit alternative folding.

Finally, the herein reported interaction of our tested sequences with Vimentin, is valuable not only from a structural point of view. Indeed, in colorectal cancer cells, TEAD4 binds the promoter of Vimentin to induce its expression and the level of these two proteins are correlated to drive EMT, ultimately increasing the migration of tumor cells and the tumor metastasis (Liu et al., 2016). Our result, by supporting a direct interaction of Vimentin with TEAD4 promoter can foresee the occurrence of a positive feedback between these two factors, that could be associated to important tumor related pathways.

In conclusion, the herein described binding interaction between Vimentin and TEAD4-full can be considered as a new target: it paves the way to the identification of novel mechanisms worth to be exploited to realize safer and more efficient treatments for oncological patients.

## Data Availability

The original contributions presented in the study are included in the article/Supplementary Material, further inquiries can be directed to the corresponding author.
