# Supplementary material for: G-quadruplexes formation within the promoter of TEAD4 oncogene and their interaction with Vimentin
Source: Front Chem. 2022 Sep 15;10:1008075. doi: 10.3389/fchem.2022.1008075 (PMC9520404; doi:10.3389/fchem.2022.1008075)
Supplement: Supplementary file 1 [file DataSheet1.docx]

Supplementary Material

# Supplementary Figures


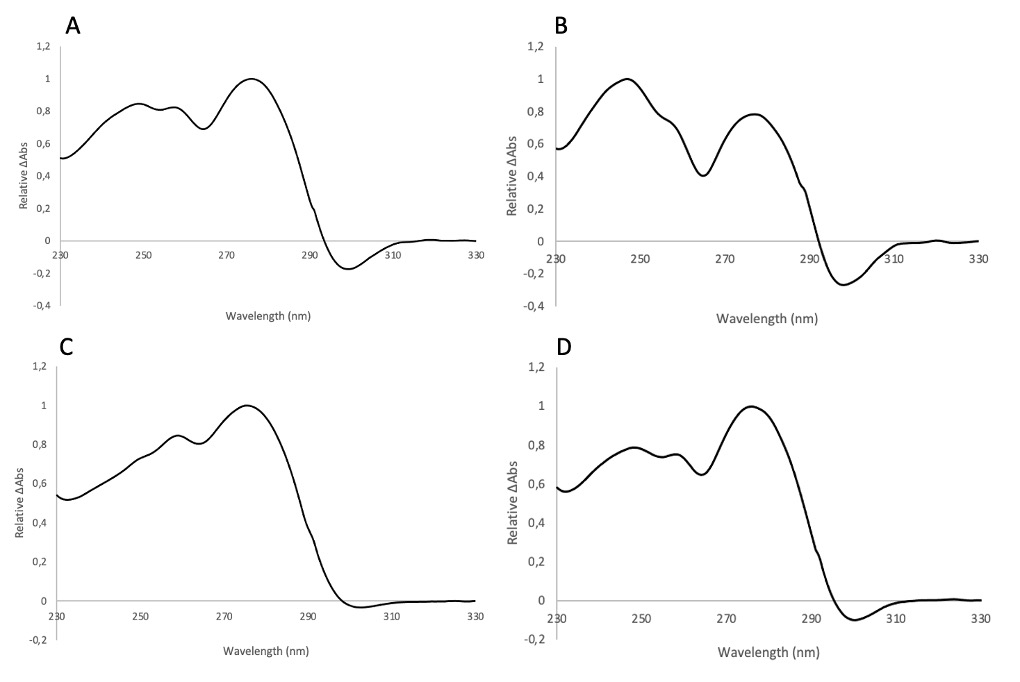


Supplementary Figure 1. TDS of (A) 4 µM TEAD4-near in 5 mM Tris pH 7.5, 150 mM KCl. (B) 4 µM TEAD4-far in 5 mM Tris, pH 7.5, 150 mM KCl. (C) and (D) 2 µM TEAD4-full in 5 mM Tris, pH 7.5, 150 mM KCl derived from spectra acquired, respectively, before and after annealing in the presence of KCl.


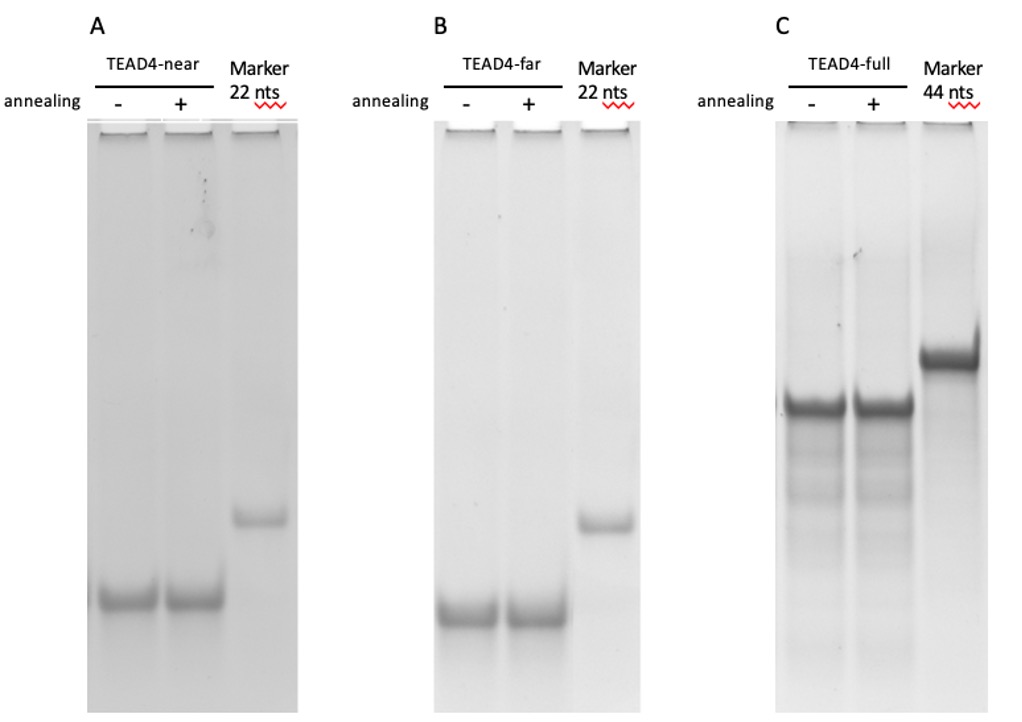


Supplementary Figure 2. Polyacrylamide gel electrophoresis of: (A) TEAD4-near; (B) TEAD4-far and (C) TEAD4-full annealed in the absence or presence of 150 mM KCl performed on 15% acrylamide in 1X TBE.

**
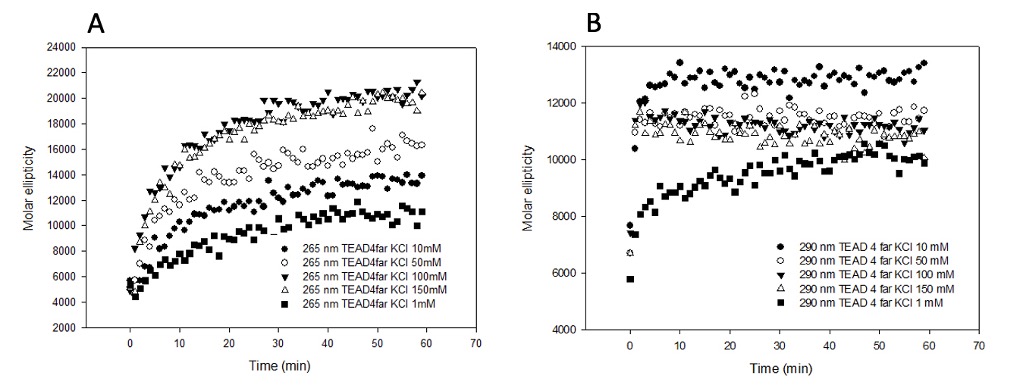
**

Supplementary Figure 3. Time dependent variation of molar ellipticity at 265 nm (A) and at 290 nm (B) of 4 µM TEAD4-far in 5 mM Tris, pH 7.5 upon addition of 1, 10, 50, 100 and 150 mM KCl.

**
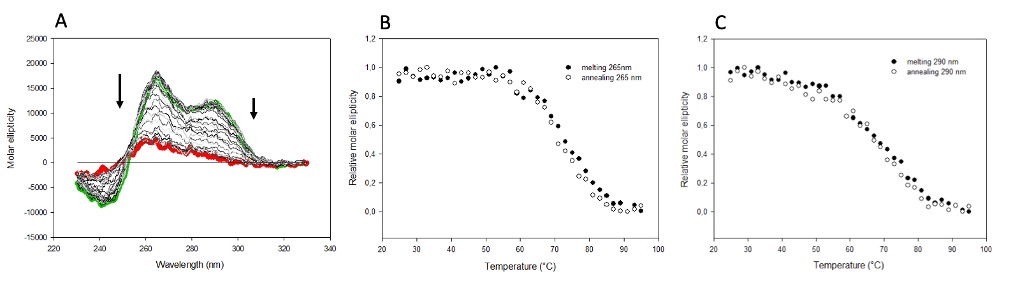
**

**Supplementary Figure 4.** (A) CD spectra of 4 µM TEAD4-far in 5 mM Tris, pH 7.5, 10 mM KCl, acquired at increasing temperatures. The green and red lines correspond to spectra acquired at 25 and 95 °C, respectively. (B) and (C) Variation of the relative molar ellipticity at 265 and 290 nm, respectively, acquired during the annealing and the melting of 4 µM TEAD4-far in 5 mM Tris, pH 7.5, 150 mM KCl.

**
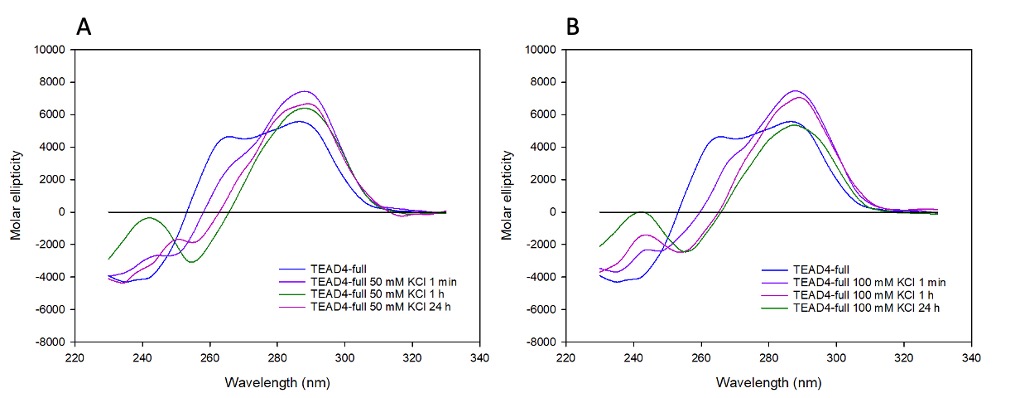
Supplementary Figure 5.** CD spectra of 2 µM TEAD4-full in 5 mM Tris, pH 7.5, in the absence of KCl and 1min, 1 h and 24 h after the addition of 50 (A) or 100 mM KCl (B) at 25 °C.

**
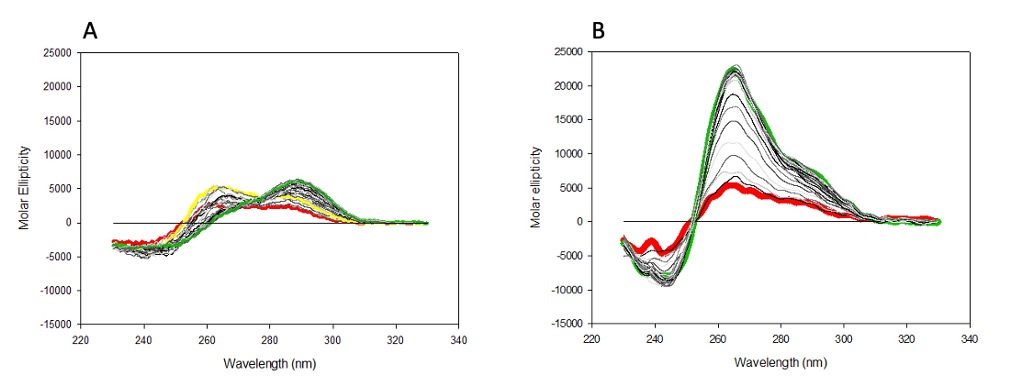
**

**Supplementary Figure 6.** CD spectra of 2 µM TEAD4-full in 5 mM Tris, pH 7.5 in 50 (A) or 150 mM KCl (B), acquired at increasing temperatures. The green, yellow and red lines correspond to spectra acquired at 25, 75 and 95 °C, respectively.

**
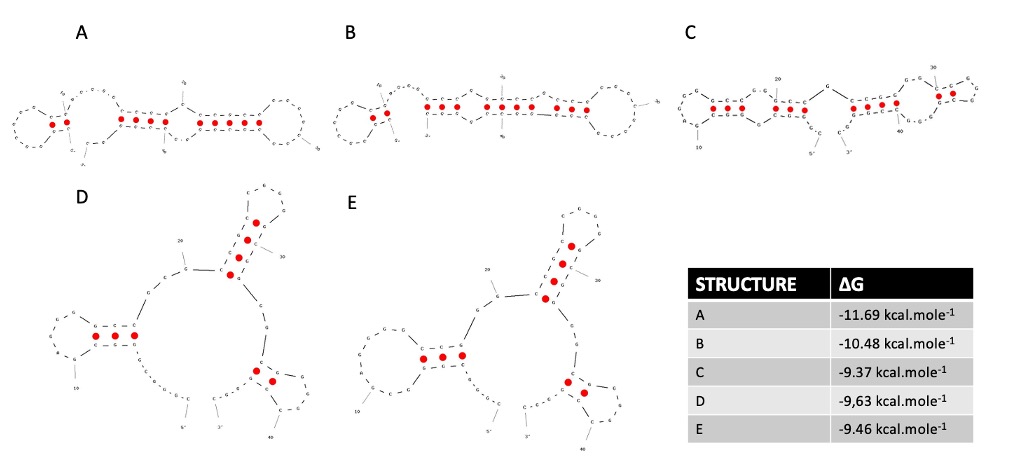
**

**Supplementary Figure 7** Prediction of hairpin formation for TEAD4-full sequence by IDT oligo analyzer.
